# Antioxidant Fucoidans Obtained from Tropical Seaweed Protect Pre-Osteoblastic Cells from Hydrogen Peroxide-Induced Damage

**DOI:** 10.3390/md17090506

**Published:** 2019-08-28

**Authors:** Gabriel Pereira Fidelis, Cynthia Haynara Ferreira Silva, Leonardo Thiago Duarte Barreto Nobre, Valquíria Pereira Medeiros, Hugo Alexandre Oliveira Rocha, Leandro Silva Costa

**Affiliations:** 1 Department of Biochemistry, Universidade Federal do Rio Grande do Norte, Natal, Rio Grande do Norte 59.078-970, Brazil; 2 Department of Biochemistry, Universidade Federal de Juiz de Fora, Juiz de Fora, Minas Gerais 36036-900, Brazil; 3 Instituto Federal de Educação, Ciência, e Tecnologia do Rio Grande do Norte (IFRN), Rio Grande do Norte, Canguaretama, Rio Grande do Norte 59.500-000, Brazil

**Keywords:** bone cells, sulfated polysaccharides, osteoporosis, brown seaweed, oxidative damage

## Abstract

Some antioxidant compounds decrease the amount of intracellular reactive oxygen species (ROS) and consequently reduce the deleterious effects of ROS in osteoblasts. Thus, these compounds fight against osteoporosis. Brown seaweeds are a rich source of antioxidant fucose-containing sulfated polysaccharides (fucans and fucoidans). We obtained six fucoidans (FRFs)—F0.3, F0.5, F0.7, F1.0, F1.5, and F2.1—from *Dictyota mertensii* by proteolytic digestion followed by sequential acetone precipitation. Except for F0.3, all FRFs showed antioxidant activity in different in vitro tests. In pre- osteoblast-like cells (MC3T3-L1) exposed to H_2_O_2_-oxidative stress, caspase-3 and caspase-9 were activated, resulting in apoptosis of the cells. We also observed a decrease in superoxide dismutase (SOD) and alkaline phosphatase (ALP) activity. The antioxidant FRFs protected the cells from the oxidative damage caused by H_2_O_2_, decreasing intracellular ROS and caspase activation, and increasing SOD activity. The most effective protection against damage was provided by F0.7, F1.5, and F2.1. At 0.5 mg/mL, these FRFs also suppressed the H_2_O_2_-mediated inhibition of ALP activity. The data indicated that FRFs F0.7, F1.5, and F2.1 from *D. mertensii* were antioxidants that protected bone tissue from oxidative stress and could represent possible adjuvants for the treatment of bone fragility through counteracting oxidative phenomena.

## 1. Introduction

Bone cells form specialized connective tissue that is responsible for the support and protection of vital organs. This tissue is composed of cells and a calcified extracellular matrix, in which there are deposits of calcium, phosphate, and other ions [1]. Bone development is a dynamic and continuous process of bone resorption and formation, mediated by differentiated osteoclasts and osteoblasts; this process is named bone remodeling, and its role is to maintain the activity of these specialized cells in harmony, as well as calcium serum levels and inorganic bone mineral density [2]. An imbalance in this homeostasis may lead to some diseases; the most common is osteoporosis, a disease that results in the reduction of bone mineral density, which increases the risk of fractures [3].

The treatment of osteoporosis is achieved using compounds that are able to inhibit bone resorption and stimulate the formation of bone tissue. The most common of the indicated therapies is hormone replacement through estrogen [4]. However, there are several side effects related to the use of these procedures over a long period, such as an increased risk of cancer, endometrial hyperplasia [5], stroke, and venous thromboembolism [6].

Therefore, new and effective treatments for osteoporosis that can minimize or even eliminate these adverse reactions from hormone replacement therapy are necessary. Osteoblasts are cells that have an important role in osteoporosis and other diseases related to bone tissue. Reactive oxygen species (ROS) are one of the main factors responsible for the deregulation of bone tissue hemostasis. When osteoblasts are under oxidative stress, ROS affect osteoblast functions, resulting in signs for induction of osteoclast differentiation. Furthermore, during severe pathologic stress, osteoblasts undergo apoptosis, which leads to an imbalance between osteoblasts and osteoclasts, and eventually to bone resorption and the onset of diseases related to this situation [7].

Recently, natural antioxidant compounds have been studied for their role in the differentiation of osteoblasts, as reactive oxygen species (ROS) are mainly responsible for the deregulation of osteoblastic activity. To this end, we have highlighted the fucose-containing sulfated polysaccharides (fucans and fucoidans) in seaweed. These molecules are acidic polysaccharides due to the presence of sulfate and/or carboxyl groups covalently bound to their structure. The fucans and fucoidans constitute a very heterogeneous group of polysaccharides whose main characteristic is their constituent sulfated L-fucose monomers [8,9]. These molecules are abundant in brown seaweed and are capable of stimulating the differentiation of mesenchymal cells into osteoblasts without toxic effects [10,11] and have antioxidant activity [12].

To the best of our knowledge, the first paper describing fucoidans with osteoblast-inducing activity was published in 2008 [13]. In this paper, the authors showed that low molecular weight fucoidans (~30 kDa) extracted from the seaweed *Ascophyllum nodosum* were able to promote the proliferation of osteoblasts, in addition to the formation of type I collagen and alkaline phosphatase (ALP), which are indicators of bone differentiation. Subsequently, a fucoidan with a much lower molecular weight (760 Da), extracted from the seaweed *Sargassum hemiphyllum*, was also able to stimulate osteogenesis [14]. However, the low molecular weight is not an important factor in the osteogenic action of fucoidans. Park et al. [15], working with a commercially available fucoidan with a molecular weight of above 30 kDa, and Kim et al. [16], working with a high molecular weight fucoidan extracted from the kelp *Laminaria japonica*, demonstrated the osteogenic effects of these compounds through the stimulation of the expression of several genes involved in osteogenesis in mesenchymal cells.

The mechanism of action of these fucoidans is related to the ability of these molecules to stimulate the proliferation of osteoblasts. We were unable to find papers that evaluated antioxidant fucoidans as osteogenic agents. In addition, the papers describing fucoidans with osteogenic activity only included fucoidans obtained from seaweed of the Fucales and Laminariales orders, which are present mainly in regions with a cold climate. The osteogenic effect of fucoidans obtained from brown seaweed found in tropical regions has not been studied. In the northeast of Brazil there are many seaweeds of the order Dictyotales, such as *Spatoglossum schröederi* [17], *Lobophora variegata* [18], *Dictyopteris justii* [19], and *Dictyota mertensii* [20]. It was demonstrated that *D. mertensii* species synthesized fucoidan with immunomodulatory action and that it was able to inhibit the growth of infective forms of *Leishmania* spp.; in addition, it was not toxic to mammalian cells [20]. It has been shown that fucoidan-rich extract of this alga, formulated with silver nanoparticles, inhibited the proliferation of tumor cells and exerted immunomodulatory and antibacterial action [21]. This extract also exhibited antioxidant activity in various in vitro tests [22]. However, no studies have evaluated the action of fucoidans obtained from these extracts as an antioxidant and osteogenic agent. Therefore, in order to indicate these fucoidans for future studies related to osteogenic differentiation our objective was to obtain fucoidan (FRFs) from *D. mertensii*, characterize these samples, and evaluate their effect as antioxidant agents, as well as protective agents on H_2_O_2_-induced oxidative damage in MC3T3 pre-osteoblastic cells.

## 2. Results

### 2.1. Physicochemical Analyses of Fucoidans (FRFs)

Six FRFs, named F0.3, F0.5, F0.7, F1.0, F1.5, and F2.1, were obtained by using an inexpensive method that combines proteolytic digestion with sequential precipitation with acetone. The data on the chemical analyses and yield of the fractionation process are shown in Table 1. As can be seen in this table, the yield ranged from 8.8% (F2.1) to 24.1% (F0.5).

With regard to the presence of proteins and sulfate in the samples, no protein was detected, and low amounts of sulfate were detected in samples F0.3 and F2.1, while an intermediate value (4.7%) was found in sample F0.5. Other fractions (F0.7, F1.0, and F1.5) had a higher sulfate presence, reaching a maximum value of 7.8%, as seen in fraction F0.7. The monosaccharide composition of the samples is also presented in Table 1; all samples contained fucose, glucose, galactose, mannose, and xylose, except for F2.1, in which xylose was not detected. It was also observed that three samples (F1.0, F1.5, and F2.1) had the main constituent of galactose, whereas F0.5 had a greater abundance of glucose.

**Figure 1:**
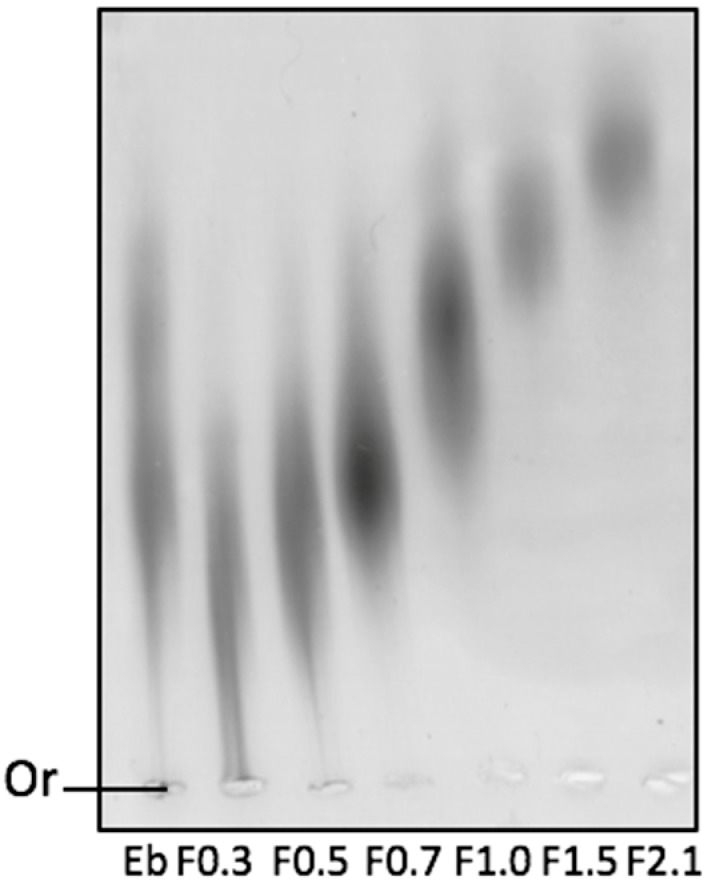
Electrophoresis in 0.05 M diaminopropane acetate buffer, pH 9.0, of fucoidans (FRFs) obtained by acetone precipitation. Approximately 5 μL (50 μg) of each FRF were applied in agarose gel prepared in diaminopropane acetate buffer and subjected to electrophoresis, as described in the methods. Or—origin; Eb—crude extract.

To confirm if the sulfate ions were covalently bound to the polysaccharides, the FRFs were loaded onto an agarose gel and exposed to an electric field containing 1,3-diaminopropane buffer. The slide containing the agarose gel was stained with toluidine blue and the electrophoretic profile of each sample was verified (Figure 1). It was observed that all samples had a negative total charge, as all samples had migrated to the positive pole. It is possible to identify that the polysaccharide samples moved differently in the agarose gel: the samples F0.3, F0.5, and F0.7 were less mobile; F1.0 and F1.5 had intermediate mobility; and F2.1 had higher electrophoretic mobility. 

### 2.2. Antioxidant Activity of FRFs

The antioxidant activities evaluated were: total antioxidant capacity (TAC), copper and ferrous chelation, superoxide radical scavenging, hydroxyl radical scavenging, and reducing power.

**Figure 2:**
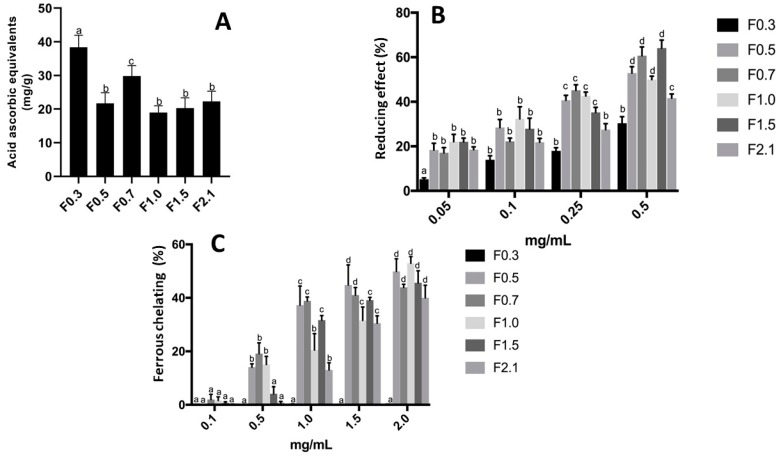
Antioxidant activities of FRFs: F0.5, F0.7, F1.0, F1.5, and F2.1 from *D. mertensii*. Total antioxidant capacity (**A**). Reducing power (**B**). Ferrous ion-chelating ability (**C**). Each value is the mean ± standard deviation of three determinations: ^a,b,c,d^ different letters indicate a significant difference (*p* < 0.05) between each concentration of the same FRF.

As can be observed in Figure 2A, antioxidant activity was detected in all samples by using the TAC assay. The values ranged from approximately 20–40 EAA/g of sample. The highest results were obtained from the F0.7 (30 EAA/g) and F0.3 (40 EAA/g) samples.

Another antioxidant activity assay performed was the reducing power activity. As shown in Figure 2B, the antioxidant activity of FRF for this assay was dose dependent. All samples had antioxidant effects at concentrations of 0.05–0.5 mg/mL, and the F0.7 and F1.5 samples showed the highest percentage in the reducing power assay, of approximately 60%.

FRFs were subjected to the chelation tests of copper and ferrous ions. For the chelation activity of copper ions, no effect of polysaccharide samples (0.1–2.0 mg/mL, data not shown) was observed. In contrast, chelation activity of the ferrous ions was observed, as shown in Figure 2C. The sample F0.3 had no chelating effect, whereas in the F2.1, ferrous chelation activity of approximately 40% at 2.0 mg/mL was detected, and in F1.5, a chelating effect of approximately 40% was detected from 1.5 mg/mL of the samples. The other samples (F0.5, F0.7, and F1.0) were able to chelate ferrous ions from a concentration of 0.5 mg/mL. However, except for F0.3, the chelating effect of the samples was not greater than 50%.

FRFs were subjected to superoxide and hydroxyl radical scavenging assays, as shown in Table 2. Only the experiments using 0.5 mg/mL F0.7 identified antioxidant activity, with approximately 10.2% for hydroxyl radical sequestration assay and 14.0% for the superoxide assay.

### 2.3. Effect of FRF on Viability of Pre-Osteoblastic Cells (MC3T3) and Osteosarcoma Cells (MG-63)

**Figure 3:**
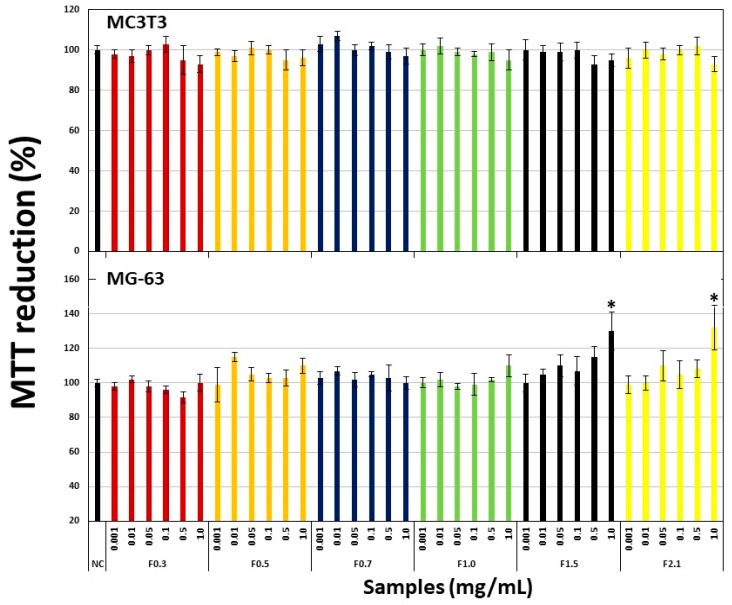
Effect of different concentrations (0.001–1.0 mg/mL) of FRFs from *D. mertensii* on the ability of MC3T3 and MG-63 cells to reduce MTT (3-(4,5-Dimethylthiazolyl-2)-2,5-diphenyltetrazolium bromide). NC—negative control, composed only of culture medium with fetal bovine serum. * *p* < 0.05 vs. NC.

The first step was to evaluate the effect of FRFs on the viability of cells obtained from bone tissue. Therefore, we chose an osteoblast-like cell line (MC3T3) and a human osteosarcoma cell line (MG-63), which are cell lines used widely in studies of osteogenic differentiation. In Figure 3, the treatment of samples did not affect the ability of the cells to reduce 3-(4,5-Dimethylthiazolyl-2)-2,5-diphenyltetrazolium bromide (MTT) regardless of the added concentration.

### 2.4. Effect of FRFs on Viability of Pre-Osteoblastic (MC3T3) Cells Exposed to Hydrogen Peroxide (H_2_O_2_).

**Figure 4:**
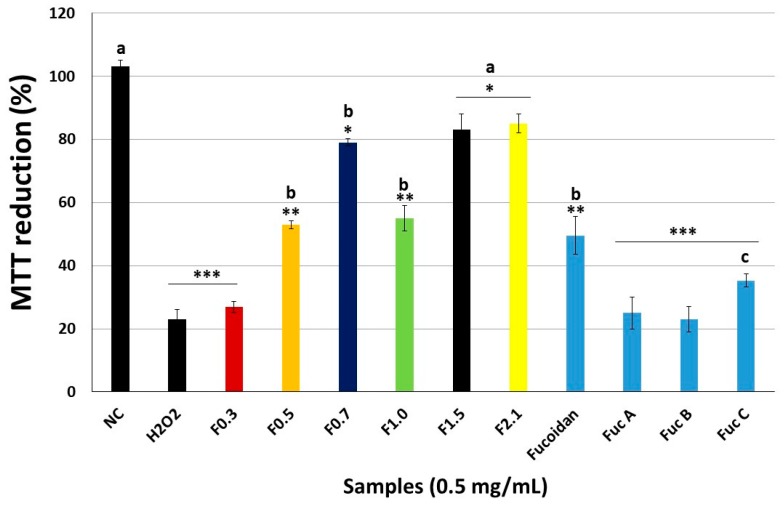
Effect of different samples (0.5 mg/mL) on the ability of pre- osteoblast-like cells (MC3T3) cells to reduce MTT in the model of oxidative stress. NC—negative control composed only of culture medium with fetal bovine serum. *** *p* < 0.001; ** *p* < 0.01; * *p* < 0.05 vs. NC. **a** *p* < 0.001; **b** *p* < 0.01; **c** *p* < 0.05 vs. H_2_O_2_.

As MC3T3 cells are commonly used in osteogenesis studies, they were chosen for further testing. In Figure 4, the effect of hydrogen peroxide (H_2_O_2_) on the ability of cells to reduce MTT is shown. In contrast to the negative control (NC), the cells exposed only to H_2_O_2_ were able to reduce just 23% of MTT reduced by the NC cells. The FRFs had different actions. F0.3 was not able to protect cells from the action of H_2_O_2_; this also occurred when it was used as three fucans of the seaweed *Spatoglossum schröederi*. F0.5 and F1.0 resulted in a moderate change, because the cells exposed to H_2_O_2_ in the presence of the drops had the capacity to reduce approximately 53% of the MTT. A similar value was obtained when the cells were exposed to fucoidan from *Fucus vesiculosus* (Figure 4). The samples of interest were F0.7, F1.5, and F2.1, because the cells exposed to these samples reduced MTT by approximately 80% compared with the NC cells

### 2.5. Intracellular Reactive Oxygen Species (ROS) Production

**Figure 5:**
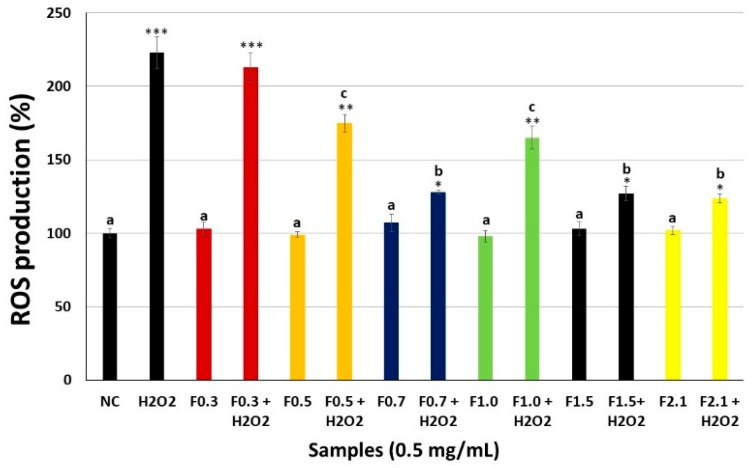
Production of reactive oxygen species (ROS) by MC3T3 cells exposed to H_2_O_2_ oxidative stress. The data presented correspond to the mean ± standard deviations (*n* = 3). NC—negative control composed only of culture medium with fetal bovine serum. *** *p* < 0.001; ** *p* < 0.01; * *p* < 0.05 vs. NC. (**a**) *p* < 0.001; (**b**) *p* < 0.01; (**c**) *p* < 0.05 vs. H_2_O_2_.

To evaluate the antioxidant effects of FRFs, we also investigated the effect of FRF on H_2_O_2_-mediated ROS production. We found that treatment with 500 μM H_2_O_2_ markedly increased ROS production. However, treatment with FRF markedly attenuated this H_2_O_2_-mediated increase intracellularly (Figure 5). The exception was F0.3, as this sample failed to decrease intracellular ROS levels after exposure of the cells to H_2_O_2_. F0.7, F1.5, and F2.1 were the most effective samples, showing the largest decrease in the amount of ROS due to the presence of H_2_O_2_.

### 2.6. Effect of FRF on Caspase-3 and Caspase-9 in MC3T3 Cells

**Figure 6:**
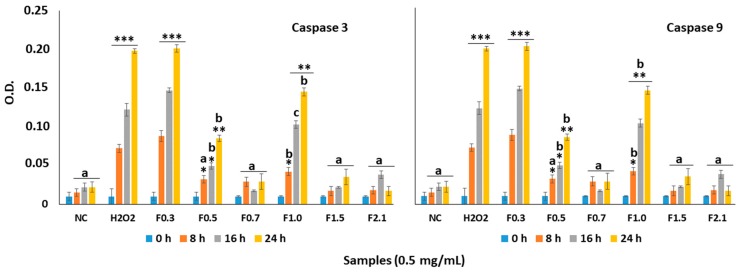
Effect of FRF (0.5 mg/mL) from *D. mertensii* on caspase-3 and caspase-9 activity in MC3T3 cells exposed to H_2_O_2_-induced oxidative stress. NC—negative control composed only of culture medium with fetal bovine serum. *** *p* < 0.001; ** *p* < 0.01; * *p* < 0.05 vs. NC. (**a**) *p* < 0.001; (**b**) *p* < 0.01; * (**c**) *p* < 0.05 vs. H_2_O_2_. O.D.—optical density.

Several studies show H_2_O_2_ induces cell death by promoting the activation of caspases. Therefore, we evaluated the activity of capase-3 and caspase-9 from the MC3T3 cells that were exposed to H_2_O_2_ in the absence and presence of FRF (0.5 mg/mL). The activity of caspase-3 and caspase-9 was very high after the cells have been exposed to oxidizing conditions (H_2_O_2_, 500 µM, final concentration), as shown in Figure 6. Moreover, the presence of FRF decreased the activity of both caspases for the various treatments.

When the effect of each sample was studied individually, the activity of the caspases in cells exposed to F0.7, F1.5, and F2.1 in the presence H_2_O_2_ was like that observed with the negative control (NC) group. In contrast, the activity of both caspases corresponded to approximately 60% to that observed in the group treated with H_2_O_2_ when the cells were exposed to F0.5 and F1.0. Only F0.3 did not protect effect against caspase activation by oxidative stress. When the cells were exposed only to FRFs, the activity of the caspases were not different those observed in NC (data not shown).

### 2.7. Assessment of the Cell Antioxidant Status

Given the pivotal role of superoxide dismutase (SOD) in limiting ROS production in oxidative stress conditions in cells, its activity was assayed in order to evaluate antioxidant balance after free radical production (Table 3). The presence of FRF (0.5 mg/mL) did not affect the SOD activity. However, a significant reduction in the levels of SOD activity was observed in experimental conditions containing H_2_O_2_, and this effect was more pronounced in the H_2_O_2_ group (without FRF).

In the presence of F0.3, the activity of SOD was similar to that found with cells treated with H_2_O_2_ only. Again, the most effective FRFs in protecting the cells from the deleterious action of H_2_O_2_ were at F0.7, F1.5, and F2.1, because in the presence of these samples the SOD activity was only 20% lower than the SOD of the NC.

### 2.8. FRFs Attenuate H_2_O_2_-Mediated Inhibition of Alkaline Phosphatase Activity (ALP) Activity

**Figure 7:**
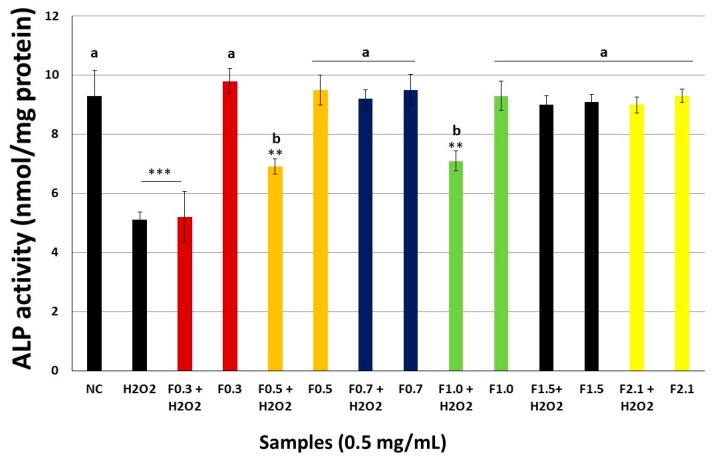
FRFs alleviated H_2_O_2_-mediated inhibition of alkaline phosphatase (ALP) activity in MC3T3 cells. The data presented correspond to the mean ± standard deviations (*n* = 3). NC—negative control composed only of culture medium with fetal bovine serum. *** *p* < 0.001; ** *p* < 0.01 vs. NC. (**a**) *p* < 0.001; (**b**) *p* < 0.01 vs. H_2_O_2_.

To investigate the effect of RFR on osteoblast differentiation, we investigated the activity of ALP in MC3T3 cells (Figure 7). Treatment with 500 μM H_2_O_2_ significantly decreased the level of ALP. However, treatment with 0.5 mg/mL FRF attenuated this effect of H_2_O_2_. Again, F0.7, F1.5, and F2.1 were the most effective samples for the protection of ALP activity of ALP suppressed by the presence of H_2_O_2_.

## 3. Discussion

In this study, we extracted six fucoidans (FRFs) from the tropical seaweed *D. mertensii*. The data in Table 1 show that these fucoidans are heteropolysaccharides. In general, algal polysaccharides of the class Dictyotales are present as well as heterogeneous compounds whose monosaccharide constitution is formed in addition to fucose by galactose, xylose, mannose, glucose, and/or glucuronic acid [23]. The other monosaccharides are often more abundant than fucose, as it is not uncommon for an individual monosaccharide to be found in greater amounts than fucose [24], as observed with the *D. mertensii* FRFs (Table 1).

The structural characteristics of fucans and fucoidans are very important to allow them to fulfil their biological function, but also define their pharmacological and biotechnological applications [25]. Therefore, the structural complexity of the fucose-containing sulfated polysaccharides, fucans, and fucoidans of the seaweeds of the Dictyotales class has attracted attention. Several of these molecules have been shown to have biological/pharmacological properties, such as the antithrombotic fucans of the seaweed *S. schröederi* [24,26], which also have antimigratory activity [27] and antiangiogenic activity [17], the antitumor fucans of the seaweed *Dictyopteris delicatula* [28], the anti-nociceptive and anti-inflammatory fucoidans of the seaweed *Dictyota menstrualis* [29], and fucans extracted from *Dictyopteris justii* seaweed that are capable of decreasing the formation of calcium oxalate crystals [19]. In addition, fucans of *Canistrocarpus cervicornis* have antioxidant activity [30].

The antioxidant activity of fucans and fucoidans was first well described by Athukorala et al. [31]. Subsequently, many other papers have shown antioxidant activity of fucans, fucoidans, extracts and fractions rich in these molecules, as can be seen in excellent reviews recently published [32,33,34]. In general, the data have shown that these compounds are good electron donors and metal chelators; however, their ability to scavenging reactive species, such as superoxide and hydroxyl ions, is lower than other antioxidant agents [12,32,33,34]. This fact was also observed with fucoidans-rich extracts obtained from different seaweeds of the class Dictyotales [22,35], as was observed with FRFs of *D. mertensii* (Figure 2).

As observed in Figure 2, the antioxidant activity of the FRFs was not similar, and, in the case of F0.3, was not detected in most tests. The factors that are related to the greater or lesser antioxidant activity of a fucoidan sample are complex; the amount of sulfate groups and how these are distributed in the molecule, the types of monosaccharides, and how these are linked together are some of the factors cited [32]. We cannot correlate the structure and activity of the fucoidans present in FRFs of *D. mertensii*. However, we were able to affirm that there was no correlation between the sulfate content of FRF and their antioxidant activities.

Antioxidant compounds are associated with the fight against oxidative stress. When the oxidative stress is uncontrolled it is associated with several pathophysiological processes, including the process of osteoclast-mediated resorption; this type of cell synthesizes free radicals for degradation of the bone matrix, and, in situations of hyperproduction or suppression of the osteoblast activity, increases bone loss by converging on osteoporosis [36].

Some fucoidans are indicated as osteoblast-inducing agents and therefore have the potential to be used in the treatment of osteoporosis [13,14,15,16]. The mechanism of action of these fucoidans is related to the ability of these molecules to stimulate the proliferation of osteoblasts. We were unable to find papers that evaluated antioxidant fucoidans as osteogenesis-inducing agents.

Antioxidants such as L-carnitine [37], and even extracts rich in antioxidants, such as porcine placenta hydrolysates [38], acted as osteogenic agents by decreasing the amount of intracellular ROS and thereby decreasing the deleterious effects of ROS in osteoblasts. However, not every antioxidant is a good osteogenic agent; for example, glutathione accelerates osteoclast differentiation and inflammatory bone destruction [39]. Consequently, and given the confirmation that the FRFs of *D. mertensii* had antioxidant activity, we evaluated their effect on cells obtained from bone tissue.

Initially, before we performed oxidative stress tests, we needed to rule out the possibility that FRFs were toxic to bone tissue. Therefore, we used two cell lines obtained from bone tissue that are also used in osteogenic differentiation studies, MC3T3 cells (pre-osteoblasts) and MG-63 cells (osteosarcoma). The MTT assay (Figure 3) showed that FRFs were not toxic to these cells. In addition, they did not stimulate bone cell proliferation as described for other fucoidans.

These two cell lines are used in osteogenic differentiation models. However, unlike MG-63, MC3T3 are not tumor-derived cells. Therefore, MC3T3 cells were chosen for the next experiments. Therefore, MC3T3 cells were subjected to a stress condition by the addition of H_2_O_2_. The data showed that the presence of H_2_O_2_ increased the amount of intracellular ROS and increased cell death (apoptosis) by activation of caspases (caspase-3 and caspase-9). In addition, the presence of H_2_O_2_ decreased SOD activity. These data corroborated with the data of other authors [38] and showed that our model was suitable for the evaluation of antioxidant compounds with osteogenic action.

The data showed that FRFs had different performances in the assay. F-0.3 was ineffective in protecting MC3T3 cells from oxidative stress induced by H_2_O_2_. This samples also presented the worst results in the antioxidant tests in vitro and was only effective on the Total Antioxidant Capacity assay; however, it was not the most potent FRF. This showed that there was a relationship between the in vitro antioxidant data and the data obtained with the cells. The other FRFs could be grouped into two groups: F0.5 and F1.0, which presented moderate action against oxidative stress, and F0.7, F1.5; and F2.1, which presented an excellent protective action against oxidative damage. As shown in Figure 4, F0.7, F1.5, and F2.1 showed a stronger effect than the other four fucose-containing sulfated polysaccharides, including the fucoidan of *Fucus vesiculosus*, which has been studied before as antioxidant agent [34,40].

From the electrophoresis slide (Figure 1), it was possible to see that the samples have a different electrophoretic profile, which indicated that each FRF contained a different type of fucoidan, partly justifying this difference in FRF activity. However, F0.3 and F0.5 presented similar electrophoretic fucoidans, showing that other characteristics were responsible for FRF activity. The discovery of these characteristics is beyond the scope of this paper, but it is hoped that future studies may bring more information on this topic.

Oxidative damage caused by H_2_O_2_ increased the activation of caspases and decreased the viability of cells (Figure 5). Data from the literature showed that increased apoptosis of osteoblasts was related to osteoporosis [41]. The FRFs, mainly F0.7, F1.5, and F2.1, increased cell viability, and decreased caspase activation in the presence of H_2_O_2_. These results demonstrated that these FRFs protected osteoblasts from apoptosis induced by oxidative stress. As the activation of caspases occurs, among several factors, by increasing the amount of intracellular ROS, we propose that FRFs inhibited apoptosis by decreasing the amount of intracellular ROS (Figure 6). FRFs also indirectly decreased the amount of ROS, as they decreased the inhibition of SOD caused by H_2_O_2_ (Table 2). As the activity of SOD increases, superoxide ions are less active, thereby decreasing the cell death induced by H_2_O_2_.

ALP is an important component in bone formation, as it acts by increasing the concentration of inorganic phosphate, an agent that promotes mineralization, but also acts to reduce the concentration of extracellular pyrophosphate, an inhibitor of mineral formation. ALP is also a primary marker of osteoblastic differentiation [42]. H_2_O_2_ promotes a decrease in the activity of this enzyme and this effect was annulled by the presence of F0.7, F1.5, and F2.1 (Table 2), indicating that these can stimulate the differentiation of osteoblasts. Other compounds, such as porcine placenta hydrolysates [38], have already been identified as agents capable of stimulating the differentiation of osteoblasts by blocking the action of H_2_O_2_ on ALP.

## 4. Materials and Methods

### 4.1. Materials

Acetonitrile, iron (II) sulfate, sulfuric acid, and potassium ferricianyde (III) were obtained from Merck (Darmstadt, Germany). Fucoidan from *Fucus vesiculosus*, ammonium molybdate, nitro Blue Tetrazolium (NBT), methionine, and monosaccharides were purchased from Sigma-Aldrich Co. (St. Louis, MO, USA). Trypsin, cell culture medium components (Dulbecco’s Modified Eagle Medium-DMEM), and newborn calf serum (FCS) were obtained from Cultilab (Campinas, SP, Brazil). Phosphate buffered saline (PBS) was puchased from Invitrogen Corporation (Burlington, ON, USA). All other solvents and chemicals were of analytical grade.

Eukaryotic cells (the murine calvaria-derived osteoblast-like cell line (MC3T3-E1) (ATCC^®^ CRL-2593^™^, Manassas, VA, USA)), and human osteosarcoma cells (MG-63 (ATCC^®^ CRL-1427^™^, Manassas, VA, USA) were grown in Dulbecco’s modified Eagle’s medium (DMEM) with 10% fetal bovine serum (FBS), 10 mg/mL streptomycin, and 10,000 IU penicillin.

The fucans from the brown seaweed *Spatoglossum schröederi*, Fuc A, Fuc B, and Fuc C were obtained as described by Amorim et al., [8]; Nobre et al., [23]; and Rocha et al., [24], respectively.

### 4.2. Extraction of Fucoidan (FRF)

The seaweed *Dictyota mertensii* was collected at Pirambuzios Beach (5°59′20.6″ S 35°06′50.1″ W), Nísia Floresta-RN, Brazil. The alga was stored in our laboratory and dried at 50 °C under ventilation in an oven, ground in a blender, and incubated with ethanol to eliminate lipids and pigments. The defatted and depigmented seaweeds were then stored in our laboratory protected from light until the extraction of the polysaccharides. The seaweed was identified according to its morphology [43]. The material collection occurred under authorization of Brazilian National Management System Genetic Heritage and Associated Traditional Knowledge (loose translation) SISGEN n° A0D4240.

About 100 g of powdered alga were suspended with five volumes (500 mL) of 0.25 M NaCl and the pH was adjusted to 8.0 with NaOH. Next, 1.5 g of Prolav 750 (Prozyn Biosolutions, São Paulo, SP, Brazil), a mixture of alkaline proteases, was added for proteolytic digestion. After incubation for 18 h at 60 °C, the mixture was filtered through cheesecloth. The resulting extract was referred to as the crude extract and it was then subjected to acetone fractionation, according to the reported method [8]. Briefly, the filtrate was fractionated by precipitation with acetone as follows: 0.3 volumes of ice-cold acetone were added to the solution under gentle agitation and maintained at 4 °C for 24 h. The precipitate formed was collected by centrifugation (10,000× *g*, 20 min), vacuum dried, resuspended in distilled water, and analyzed. The operation was repeated by adding 0.5, 1.0, and 2.0 volumes of acetone to the supernatant. Based on the acetone volumes used in the fractionation step, the obtained fractions were dominated by F0.3, F0.5, F0.7, F1.0, F1.5, or F2.0.

### 4.3. Chemical Analysis and Monosaccharide Composition

Sulfate content was determined according to the gelatin-barium method [44], using sodium sulfate (1 mg/mL) as standard and after acid hydrolysis of the polysaccharides (4 M HCl, 100 °C, 6 h). Protein content was measured using Spector’s method [45]. The polysaccharides were hydrolyzed with 0.5, 1, 2, and 4 M, respectively, for various lengths of time, (0.5, 1, 2 and 4 h), at 100 °C. Reducing sugars were determined using the Somogyi–Nelson method [46]. After acid hydrolysis, sugar composition was determined by a LaChrom Elite^®^ HPLC system with a refractive index detector (RI detector model L-2490) (Hitachi Ltd., Tokyo, Japan). A LichroCART^®^ 250-4 column (250 mm × 40 mm) packed with Lichrospher^®^ 100 NH2 (5 µm) (Hitachi Ltd., Tokyo, Japan) was coupled to the system. The sample mass used was 0.2 mg and analysis time was 25 min. The following sugars were analyzed as references: arabinose, fructose, fucose, galactose, glucose, glucosamine, glucuronic acid, mannose, and xylose.

### 4.4. 3-(4,5-Dimethylthiazolyl-2)-2,5-diphenyltetrazolium Bromide (MTT) Test

For the tests, 0.5 × 10^4^ cells were grown in 96-well plates with DMEM medium containing the samples in concentrations from 0.001 to 1 mg/mL for 24 h (each concentration in triplicate). The cell capacity to reduce MTT was determined by the colorimetric test of MTT as described earlier [8].

### 4.5. Agarose Gel Electrophoresis in 1,3-Diamino Propane Acetate Buffer (PDA)

The electrophoretic mobility of *D. mertensii* FRF was evaluated by gel electrophoresis in PDA buffer, according to the reported method [47]. Initially, glass slides were coated with 0.6% (*m/v*) agarose in PDA buffer (0.05 M, pH 9.0). Subsequently, aliquots of the polysaccharides (about 50 μg) were applied to the gel and subjected to electrophoresis (100 V, 4 °C) for 60 min. After the electrophoretic run, the polysaccharides were precipitated with 0.1% cetyltrimethylammonium bromide (CETAVLON, Sigma Chemical Company, St. Louis, MO, USA) for 2 h at room temperature and the gels were dried using warm air stream. To visualize the SPs, gels were stained with a solution of 0.1% toluidine blue in 1% acetic acid and 50% ethanol. The gel was then de-stained with the same solution without the dye. Three independent analyses were performed.

### 4.6. Antioxidant Activity

Five assays were performed to analyze the antioxidant activity of the sulfated polysaccharides obtained: total antioxidant capacity, hydroxyl radical scavenging, superoxide radical scavenging, reducing power assay, and ferric chelating, as previously described [19,48].

#### 4.6.1. Determination of Total Antioxidant Capacity (TAC)

This assay is based on the reduction of Mo (VI) to Mo (V) by sulfated polysaccharides and subsequent formation of a green phosphate/Mo(V) complex at acid pH. Tubes containing sulfated polysaccharides and reagent solution (0.6 M sulfuric acid, 28 mM sodium phosphate and 4 mM ammonium molybdate) were incubated at 95 °C for 90 min. After the mixture had cooled to room temperature, the absorbance of each solution was measured at 695 nm against a blank. Total antioxidant capacity was expressed as ascorbic acid equivalent.

#### 4.6.2. Hydroxyl Radical Scavenging Activity Assay

The scavenging activity of seaweed polysaccharides against the hydroxyl radical was investigated using Fenton’s reaction (Fe^2+^ + H_2_O_2_ → Fe^3+^ + OH^−^ + OH). These results were expressed as inhibition rate. Hydroxyl radicals were generated using 3 mL sodium phosphate buffer (150 mM, pH 7.4), which contained 10 mM FeSO_4_.7H_2_O, 10 mM EDTA, 2 mM sodium salicylate, 30% H_2_O_2_ (200 mL), and varying polysaccharide concentrations. In the control, sodium phosphate buffer replaced H_2_O_2_. The solutions were incubated at 37 °C for 1 h, and the presence of the hydroxyl radical was detected by monitoring absorbance at 510 nm. Gallic acid was used as positive control.

#### 4.6.3. Ferrous Ion-Chelating Ability

The chelating ability of samples was evaluated as described by Wang et al. [49]. Briefly, each sample at different concentrations (from 0.5 to 0.5) was added to the reaction mixture containing FeCl_2_ (0.05 mL, 2 mM) and ferrozine (0.2 mL, 5 mM). The mixture was shaken and incubated for 10 min at room temperature and absorbance of the mixture was measured (562 nm) against a blank (ultrapure water). Control was the reaction mixture without polysaccharides.

The chelating effect was calculated using the corresponding absorbance (A) in the formula given below, where control is the absorbance in the absence of chelating agents:

(1) $$\mathrm{Chelating}\;\mathrm{Effect}\;\left(\%\right)=\left(\frac{A_{\mathrm{control}}-A_{\mathrm{sample}}}{A_{\mathrm{control}}}\right)*100$$

#### 4.6.4. Copper Chelating

The ability to chelate the copper ion from the extracts was determined by the method described by Anton [50]. Pyrocatechol violet, the reagent used in this assay, can associate with certain cations such as aluminum, copper, bismuth, and thorium. In the presence of chelating agents this combination is not formed, resulting in decreased staining. This reduction thus allows for estimating the chelating activity of the copper ion from the fucoidans. The test is performed in 96-well microplates with a reaction mixture containing different concentrations of samples (0.1–20 mg/mL), pyrocatechol violet (4 mM), and copper II sulfate pentahydrate (50 mg/mL). All wells were homogenized with the aid of a micropipette and the solution absorbance was measured at 632 nm. The chelating effect was calculated using the corresponding absorbance in the formula given below, where blank is the absorbance in the absence of chelating agents.

(2) $$\mathrm{Chelating}\;\mathrm{Effect}\;\left(\%\right)=\left(\frac{A_{\mathrm{blank}}-A_{\mathrm{sample}}}{A_{\mathrm{blank}}}\right)*100$$

#### 4.6.5. Superoxide Radical Scavenging Activity Assay

This assay was based on the capacity of sulfated polysaccharides to inhibit the photochemical reduction of NBT in the riboflavin–light–NBT system. Each 3 mL of reaction mixture contained 50 mM phosphate buffer (pH 7.8), 13 mM methionine, 2 mM riboflavin, 100 mM EDTA, NBT (75 mM), and 1 mL sample solution. After the production of blue formazan, the increase in absorbance at 560 nm after 10 min illumination from a fluorescent lamp was determined. The entire reaction assembly was enclosed in a box lined with aluminum foil. Identical tubes with the reaction mixture were kept in the dark and served as blanks. Gallic acid was used as positive control.

#### 4.6.6. Reducing Power

The reducing power assay depends on the reduction of the potassium ferricyanide by the samples. Briefly, the FRFs were mixed with a phosphate buffer 0.2 M (pH 6.6) and potassium ferricyanide (1% *m*/*v*) and incubated at 50 °C for 20 min. One addition of trichloroacetic acid (10% *m*/*v*) was used to in order to stop the reaction. Distilled water and ferrous chloride (0.1% *m*/*v*) were added to the solution and the absorbing capacities were measured at 700 nm. Results were calculated as an activity percentage, considering the largest concentration of ascorbic acid (the standard) as 100% activity.

### 4.7. Induced Oxidative Stress Assay

MC3T3 cells (1 × 10^6^ cells/mL) were placed in 6-well plates in the presence of 1 mL of DMEM supplemented with 10% FCS. After 24 h, the plates were washed and 1 mL of DMEM supplemented with 10% FCS and sulfated polysaccharides (from 0.001 to 1 mg/mL) and H_2_O_2_ (500 µM, final concentration) were added. The plates were kept in culture condition (37 °C; 5% CO_2_; dark) for 6 h; thus, the medium was replaced by 1 mL of the same fresh medium. After 24 h, the cells were submitted to MTT test as described above.

### 4.8. Caspase-3 and -9 Activity Assays

MC3T3 cells (1 × 10^6^ cells/mL) were placed in 6-well plates in de presence of 1 mL of DMEM supplemented with 10% FCS. After 24 h, the plates were washed with 1 mL of DMEM supplemented with 10% FCS, sulfated polysaccharides (0.5 mg/mL) or sulfated polysaccharides (0.5 mg/mL) and H_2_O_2_ (500 µM, final concentration). After 6 h, the medium was replaced by fresh medium. At 8, 16, and 24 h the plates were washed with ice-cold PBS and scraped into 200 mL of lysis buffer (50 mM Tris-HCl (pH 7.4), 1% Tween 20, 0.25% sodium deoxycholate, 150 mM NaCl, 1 mM EDTA, 1 mM Na_3_VO_4_, 1 mM NaF), and protease inhibitors (1 mg/mL aprotinin, 10 mg/mL leupeptin and 1 mM 4-(2-aminoethyl) benzenesulfonyl fluoride) for 2 h in ice. The same conditions were used for untreated cells in the 0, 8, 16, and 24 h. Protein extracts were cleared by centrifugation and protein concentrations were determined using Bradford reagent [48] with bovine serum albumin as standard. In vitro caspase-3 and -9 protease activity was measured using a caspase activation kit according to the manufacturer’s protocol (Invitrogen, São Paulo, Brazil). For this, 50 µL of cell lysate were mixed with 50 µL of 2× reaction buffer (containing 10 µL of 1 M dithiothreitol and 5 µL of 4 mM synthetic tetrapeptide Asp-Glue-Val (for caspase 3) or Leu-Glu-His-Asp (for caspase 9) conjugated top-nitroanilide (pNA)) in a 96-well plate, after which the mixture was incubated for 2 h at 37 °C in the dark. Active caspase cleaves the peptide and releases the chromophore pNA that can be detected spectrophotometrically at a wavelength of 405 nm. Theoretically, the apoptotic cell lysates containing active tested caspases should yield a considerable emission compared with the non-apoptotic cell lysates. Data presented are representative of those obtained in at least three independent experiments done in triplicates.

### 4.9. Superoxide Dismutase Evaluation

The SOD activity was measured using commercially available kit (SOD activity Enzo Life Sciences, Farmingdale, NY, USA). The principle of the method is based on the ability of SOD to neutralize superoxide ions created by the xanthine/xanthine oxidase system and subsequently inhibit the reduction of WST-1 (water soluble tetrazolium salt) to WST-1 formazan. Briefly, MC3T3 cells (5 × 10^6^ in six-well plate), obtained 6 h after oxidative stress induction, were washed with ice-cold 1× PBS, and lysed as described in kit protocol. The supernatant of each sample was collected, and the total SOD activity was assayed spectrophotometrically at 450 nm.

### 4.10. Production of Intracellular ROS

The levels of intracellular ROS were evaluated by quantifying the fluorescence emitted by 2′,7′-dichlorofluorescein, the oxidized form of 2′,7’-dichlorofluorescein diacetate (DCFH-DA). For this purpose, MC3T3 cells were cultured were exposed to same conditions described in topic 4.8. At the end of the treatment period, the supernatant was removed, cells were washed with phosphate buffered saline (PBS), and 100 μM DCFH-DA in DMEM containing 1% FBS were added and subsequently incubated at 37 °C for 2 h. Not incorporated DCFH was then removed, cells were washed three times with PBS, and the emitted fluorescence was quantified on a flow cytometer (FACSCanto II; BD Biosciences, Eugene, OR, USA). The results were analyzed in FlowJo software (FlowJo, Ashland, OR, USA) and expressed as percent (%) of fluorescence emitted relative to control.

### 4.11. Measurement of Alkaline Phosphatase Activity

Alkaline phosphate (ALP) activity was measured for screening the osteogenic effect of each FRF sample on MC3T3. Cells (1 × 10^6^ cells/mL) were placed in 6-well plates in the presence of 1 mL of DMEM supplemented with 10% FCS. After 24 h, the plates were washed and 1 mL of DMEM supplemented with 10% FCS, H_2_O_2_ (500 µM, final concentration) and/or sulfated polysaccharides (0.5 mg/mL) was added. After 6 h, cells were lysed. The ALP activity was accessed using p-nitrophenyl phosphate (Sigma-Aldrich Co.) as described by Chaves et al. [51]. ALP activity was normalized by total cellular protein. The ALP activity assay was performed in triplicate in three independent experiments.

### 4.12. Statistical Analysis

All data were expressed as mean ± standard deviation (*n* = 3) of three observation. Statistical analysis was done by one-way ANOVA followed by the Turkey–Kramer test or Student–Newman–Keuls test. All tests were conducted on the SigmaPlot^®^ (Systat software, San Jose, CA, USA). In all cases, statistical significance was set at *p* < 0.05.

## 5. Conclusions

We obtained six antioxidant fucoidan from the brown alga *D. mertensii.* These samples, mainly comprising fucoidans, were designated F-0.3, F-0.5, F-0.7, F-1.0, F-1.5, and F-2.1. None of the samples exhibited cytotoxicity on MC3T3 cell. In addition, five samples showed antioxidant activity in various in vitro assays. Five samples assessed protected the cells from oxidative damage. F-0.7, F-1.5, and F-2.1 provided the most effective protection to MC3T3 cells against H_2_O_2_-induced stress, through the suppression of ROS production and the induction of apoptosis by ROS. Furthermore, these samples regulated oxidative stress-inhibited ALP activity. Overall, our data suggested that F-0.7, F-1.5, and F-2.1 may have potential therapeutic value for the treatment of bone formation disturbances.

Our aims for future studies include the identification of other mechanisms involved in mitigation of oxidative stress caused by H_2_O_2_ and the role of sulfated polysaccharides in the mitigation of this stress. In addition, as F-0.7, F-1.5, and F-2.1 showed notable protective activity, we intend to assess their effect in vivo to identify their role as a putative drug to be used for the treatment of diseases associated with bone formation disturbances.

## Figures and Tables

**Table 1:** Chemical composition of fucoidans F0.3, F0.5, F0.7, F1.0, F1.5, and F2.1 from the brown seaweed *Dictyota mertensii*.

FRF	Yield (%)	Proteins (%)	Sulfate Content (%)	Molar Ratio				
				Fuc	Glu	Man	Gal	Xyl
F0.3	19.1	nd	1.50	1.00	0.70	0.30	1.46	0.22
F0.5	24.1	0.06	4.70	1.00	2.02	1.02	1.79	0.49
F0.7	18.2	0.04	7.80	1.00	0.24	0.26	1.78	0.68
F1.0	8.9	0.02	6.30	1.00	1.23	1.01	5.01	1.80
F1.5	21.1	0.00	6.40	1.00	2.11	0.68	4.10	0.71
F2.1	8.8	0.00	1.20	1.00	1.56	0.00	7.26	0.52

Fuc—fucose, Glu—glucose, Man—mannose, Gal—galactose, Xyl—xylose. Nd—not detected. All samples obtained by acetone precipitation were dried and weighed, and the total polysaccharides corresponded to 100%.

**Table 2:** Antioxidant activity, as determined by the hydroxyl radical scavenging and superoxide radical scavenging assays in the presence of FRF of *D. mertensii*.

	Concentration (mg/mL)	Scavenging %	
		OH.	O_2_
F0.3	0.5	0.0 ± 0.0	0.0 ± 0.0
F0.5	0.5	1.2 ± 1.7	0.5 ± 1.1
F0.7	0.5	9.0 ± 1.4 *	10.7 ± 1.7 *
F1.0	0.5	0.2 ± 0.9	1.0 ± 1.7
F1.5	0.5	0.9 ± 2.3	1.3 ± 2.0
F2.1	0.5	0.0 ± 0.0	0.0 ± 0.0

Each value represents the percentage scavenging of these radicals for FRF concentration of 0.5 mg/mL. The asterisk (*) indicates significant radical scavenging activity analyzed by one-way ANOVA followed by the Student–Newman–Keuls test (*p* < 0.05).

**Table 3:** Evaluation of the protective effect of FRFs on the MC3T3 cells exposed to oxidative damage.

Effect of FRF (0.5 mg/mL) on Total Superoxide Dismutase Levels (% of Control) of MC3T3 Cells Exposed to Oxidative Damage			
.	.	FRF	FRF + H_2_O_2_
NC	100 ± 1.1%		
H_2_O_2_	52.2 ± 1.1% ***		
F0.3		101.8 ± 2.1%	53.9 ± 0.5% ***
F0.5		101.9 ± 1.0%	74.7 ± 1.1% **
F0.7		104.2 ± 2.4%	88.8 ± 2.5% *
F1.0		105.8 ± 3.7%	52.9 ± 2.8% ***
F1.5		99.7 ± 0.7%	86.3 ± 2.8% *
F2.1		95.0 ± 3.7%	87.0 ± 0.8% *

NC—negative control (culture medium with fetal bovine sérum). *** *p* < 0.001, ** *p* < 0.01; * *p* < 0.05 vs. NC.
